# Supplementary material for: Stratospheric water vapor affecting atmospheric circulation
Source: Nat Commun. 2023 Jul 3;14:3925. doi: 10.1038/s41467-023-39559-2 (PMC10318029; doi:10.1038/s41467-023-39559-2)
Supplement: Supplementary file 1 — Supplementary information [file 41467_2023_39559_MOESM1_ESM.pdf]

Supplementary information: Stratospheric water vapor affecting atmospheric circulation

Edward Charlesworth[1*†], Felix Plöger[1,2†], Thomas Birner[3], Rasul Baikhadzhaev[1], Marta Abalos[4], Nathan Luke Abraham[5,6], Hideharu Akiyoshi[7], Slimane Bekki[8], Fraser Dennison[9], Patrick Jöckel[10], James Keeble[5,6], Doug Kinnison[11], Olaf Morgenstern[12], David Plummer[13], Eugene Rozanov[14], Sarah Strode[15,16], Guang Zeng[12], Tatiana Egorova[14], Martin Riese[1]

[1*]Institute for Energy and Climate Research: Stratosphere (IEK–7), Research Center Jülich, Jülich, Germany.
[2]Institute for Atmospheric and Environmental Research, University of Wuppertal, Wuppertal, Germany.
[3]Meteorological Institute Munich, Ludwig Maximilians University of Munich, Munich, Germany.
[4]Earth Physics and Astrophysics Department, Universidad Complutense de Madrid, Madrid, Spain.
[5]National Centre for Atmospheric Science (NCAS), University of Cambridge, Cambridge, UK.
[6]Yusuf Hamied Department of Chemistry, University of Cambridge, Cambridge, UK.
[7]National Institute for Environmental Studies, Tsukuba, Japan.
[8]Laboratoire de Météorologie Dynamique (LMD/IPSL), Palaiseau, France.
[9]Commonwealth Scientific and Industrial Research Organization (CSIRO) Environment, Aspendale, Vic. 3195, Australia.
[10]Institut für Physik der Atmosphäre, Deutsches Zentrum für Luft- und Raumfahrt (DLR), Oberpfaffenhofen, Germany.
[11]Atmospheric Chemistry Observations and Modeling Laboratory, National Center for Atmospheric Research, Boulder, CO 80301, USA.
[12]National Institute of Water and Atmospheric Research, Wellington, New Zealand.

[13]Climate Research Branch, Environment and Climate Change Canada, Montreal, Canada.
[14]Physikalisch-Meteorologisches Observatorium, Davos World Radiation Center, Davos Dorf, Switzerland.
[15]Goddard Earth Sciences Technology and Research (GESTAR-II), Morgan State University, Baltimore, MD, 21251, USA.
[16]NASA Goddard Space Flight Center, Greenbelt, MD 20771, USA.

*Corresponding author(s). E-mail(s): e.charlesworth@fz-juelich.de;
Contributing authors: f.ploeger@fz-juelich.de; thomas.birner@physik.uni-muenchen.de; r.baikhadzhaev@fz-juelich.de; mabalosa@ucm.es; N.Luke.Abraham@ncas.ac.uk; hakiyosi@nies.go.jp; Slimane.Bekki@latmos.ipsl.fr; fraser.dennison@csiro.au; Patrick.Joeckel@dlr.de; james.keeble@atm.ch.cam.ac.uk; dkin@ucar.edu; olaf.morgenstern@niwa.co.nz; david.plummer@ec.gc.ca; e.rozanov@pmodwrc.ch; sarah.a.strode@nasa.gov; Guang.Zeng@niwa.co.nz; tatiana.egorova@pmodwrc.ch; m.riese@fz-juelich.de;
†These authors contributed equally to this work.

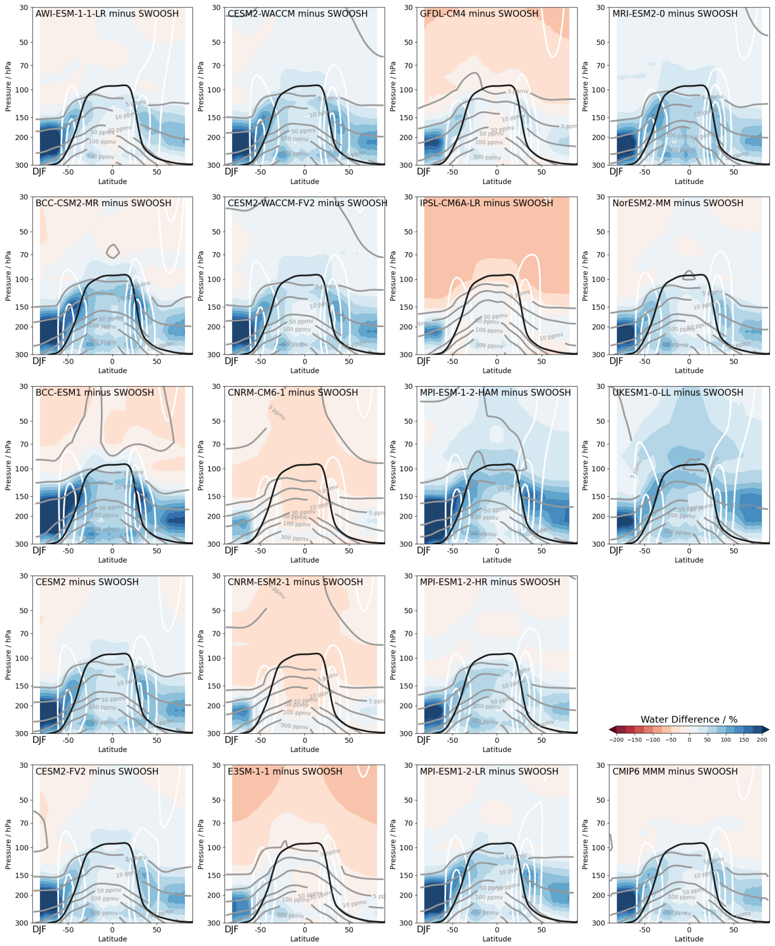

Supplementary Figure 1 Lowermost stratosphere moist bias in individual CMIP6 climate model simulations. Relative differences in water vapor distributions compared to SWOOSH merged satellite observations for CMIP6 multi model mean (lower right) and individual CMIP6 models (all other panels) for boreal winter (December–February). Relative differences are calculated by the relevant distribution minus SWOOSH as a percentage of local SWOOSH values. Only data from 2000–2014 were used. The SWOOSH water vapor distribution is shown in light grey solid contours, the 20, 30, and 40 m/s zonal wind contours from the relevant distributions are shown in white, and the tropopause as thick black line.

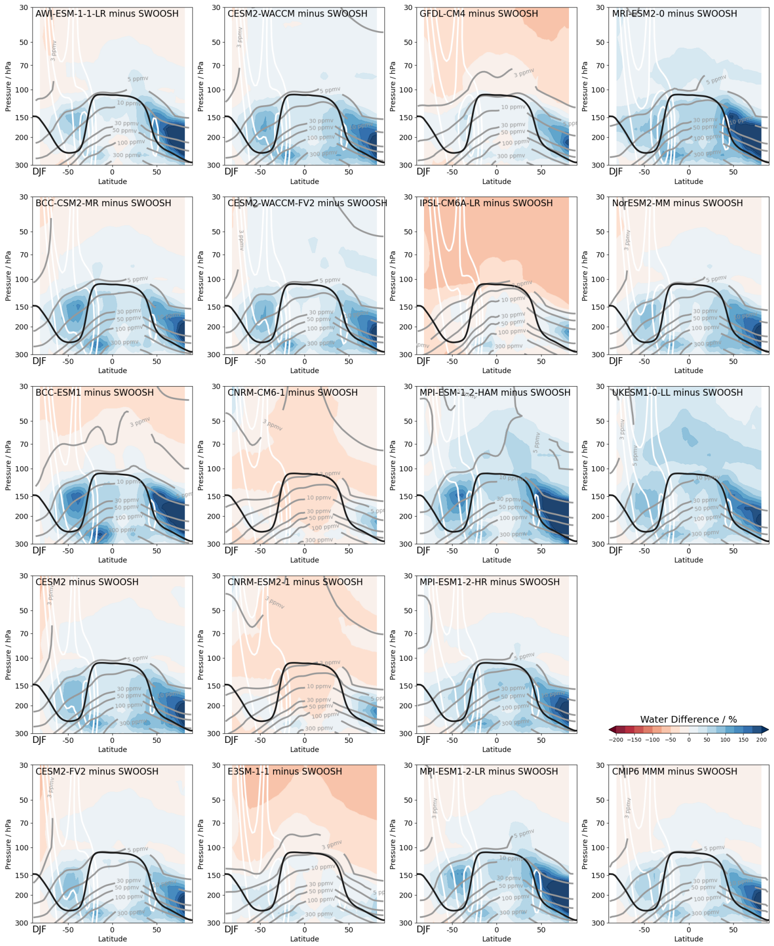

Supplementary Figure 2 Lowermost stratosphere moist bias in CMIP6 model simulations, boreal summer. Same as Figure ??, but for boreal summer (June–August).

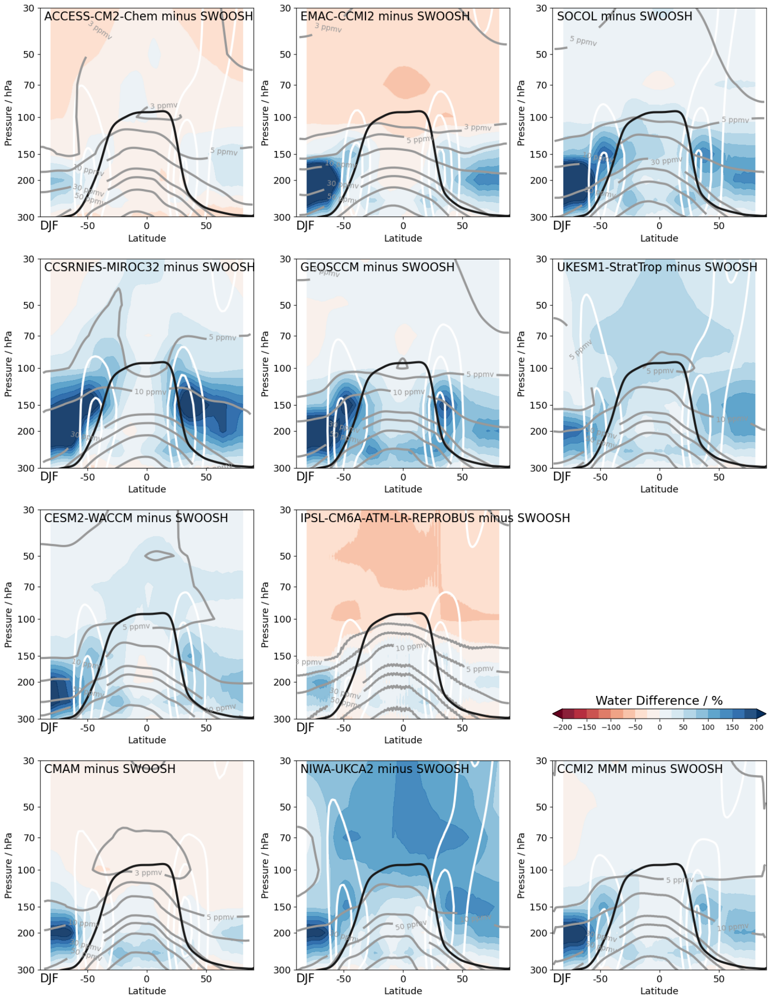

Supplementary Figure 3 Lowermost stratosphere moist bias in individual CCMI2 climate model simulations. Relative differences in water vapor distributions compared to SWOOSH merged satellite observations for CCMI2 multi model mean (lower right) and individual CCMI2 models (all other panels) for boreal winter (December–February). Relative differences are calculated by the relevant distribution minus SWOOSH as a percentage of local SWOOSH values. Only data from 2000–2018 were used. The SWOOSH water vapor distribution is shown in light grey solid contours, the 20, 30, and 40 m/s zonal wind contours from the relevant distributions are shown in white, and the tropopause as thick black line.

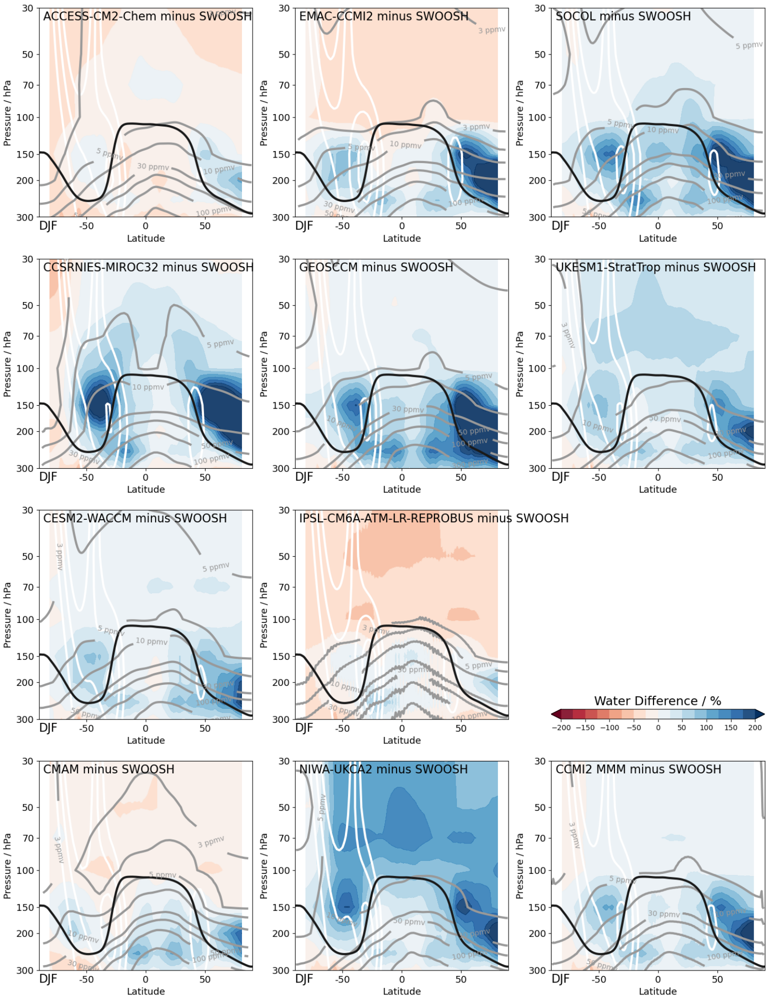

Supplementary Figure 4 Lowermost stratosphere moist bias in CCMI2 model simulations, boreal summer. Same as Figure **??**, but for boreal summer (June–August).

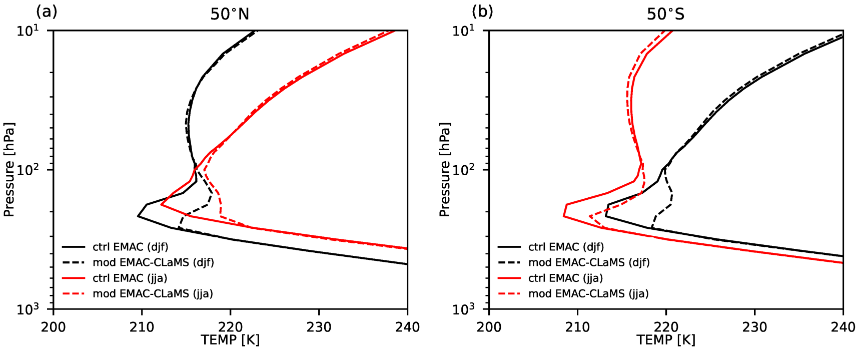

Supplementary Figure 5 Changes in tropopause height induced by lowermost stratospheric water vapor. (a) Climatological temperature profiles at 50°N are shown for boreal winter (December–February, black) and summer (June–August, red) from control (solid lines) and Lagrangian modified (dashed) EMAC simulations. (b) Shows the same, but at 50°S.

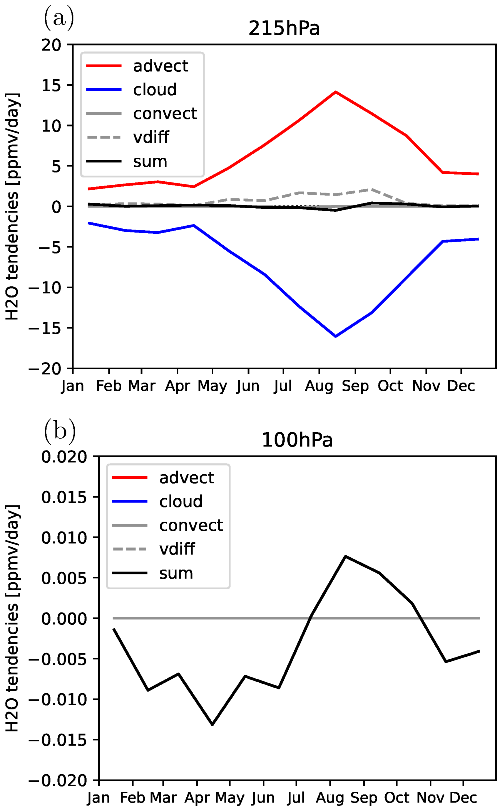

Supplementary Figure 6 Tendency contributions to lowermost stratospheric water vapor. Tendencies for control EMAC simulated water vapour at (a) 215 hPa and (b) 100 hPa averaged over the middle latitude region (50°-70°N). Shown is the seasonal cycle of tendencies for advection, cloud processes (including e.g. dehydration, evaporation, ice sublimation), convection, parameterized vertical diffusion, and the sum of all these individual contributions. At 100 hPa, the tendencies due to convection, clouds and vertical diffusion overlie the zero line.
